# Primary Langerhans Cell Histiocytosis of the Extrahepatic Bile Duct Occurring in an Adult Patient

**DOI:** 10.4274/balkanmedj.2017.1730

**Published:** 2018-11-15

**Authors:** Ifeyinwa E. Obiorah, Alicia Henao Velasquez, Bhaskar Kallakury, Metin Özdemirli

**Affiliations:** 1 Department of Pathology, Medstar Georgetown University Hospital, Washington, USA

**Keywords:** Bile duct, histiocytosis, Langerhans cell, sclerosing cholangitis

## Abstract

**Background:**

Langerhans cell histiocytosis is characterized by an abnormal proliferation of neoplastic Langerhans cells. Langerhans cell histiocytosis commonly affects the pediatric population, whereas presentation in adults remains a rare event. The presentation of Langerhans cell histiocytosis is highly variable, but the involvement of skin, bone, and lung is very common. Langerhans cell histiocytosis presenting as a bile duct mass is rare and usually occurs as part of a multiorgan system disease.

**Case Report:**

We present a case of Langerhans cell histiocytosis confined to the extrahepatic bile duct in a 62-year-old female patient with sclerosing cholangitis. The mass was composed of mononuclear cells with cleaved nuclei that were positive for CD68, S100, and CD1a as assessed by immunohistochemistry.

**Conclusion:**

This is the first report of Langerhans cell histiocytosis limited to the extrahepatic bile duct in an adult patient. We discuss the clinical manifestations and the challenges encountered in the diagnosis and treatment of this rare entity.

Langerhans cell histiocytosis (LCH), formerly known as histiocytosis X, is a wide spectrum of clinical disorders involving abnormal accumulation of Langerhans cells in various organs. Langerhans cells are derived from the dendritic cells, which are typically found in the epidermis and function as antigen-presenting cells to the immune system. Proliferation of abnormal Langerhans cells, which are closely related to histiocytes, in either single-organ or multiorgan system can result in organ dysfunction (1). Depending on the extent of involvement, LCH can result in benign or life-threatening morbid outcomes (2). LCH is a rare disease and is primarily observed in pediatric patients, with 50%-90% of cases being diagnosed between the ages of 1 and 15 years (2,3). Adults are rarely affected. The clinical manifestation of LCH is heterogeneous, based on which it can be classified as single organ or multiorgan system disease. The involvement of skin, lungs, and bone is common in single organ disease. Liver and bile duct involvement is usually observed in the disseminated form of LCH. Primary LCH of the extrahepatic bile duct is a rare event and, to date, only one case has been described in the pediatric population. To the best of our knowledge, LCH confined to the bile duct has never been reported in an adult patient. Here we describe a unique presentation of LCH presenting as a solitary lesion limited to the extrahepatic bile duct in an adult female patient.

## CASE PRESENTATION

**Figure 1:**
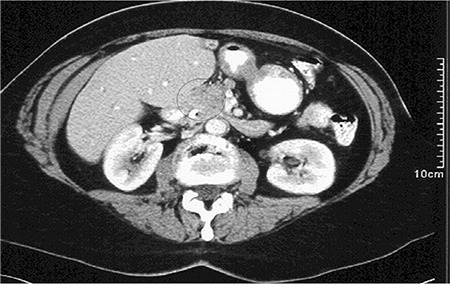
Computed tomography scan of the bile duct mass. The image shows an enhancing mass in the porta hepatis.

**Figure 2:**
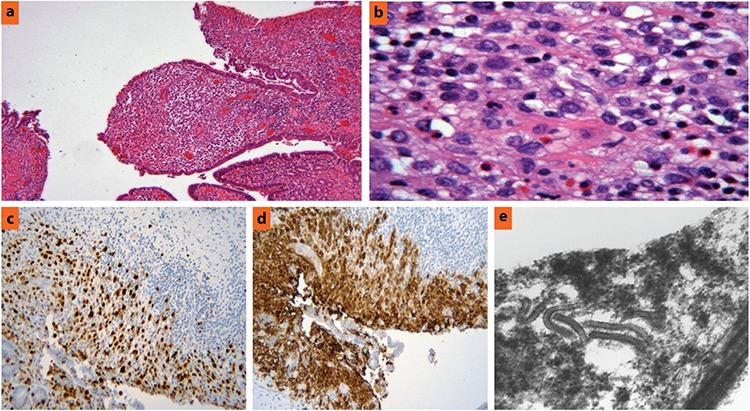
a, e. Pathologic examination of the biliary mass. Mononuclear bean-shaped cells with cleaved nuclei and abundant cytoplasm admixed with eosinophils infiltrating the bile duct. Hematoxylin and eosin ×40 (a), Hematoxylin and eosin ×400 (b), Immunohistochemistry revealed atypical cells positive for S100 (c) and CD1a (×200 each) (d), Electron microscopy of the infiltrating cells showed Birbeck granules in the cytoplasm (e).

A 62-year-old female patient was referred to our institution for further diagnostic workup of elevated liver enzymes and incidental cholelithiasis. She complained of worsening jaundice, nausea and vomiting, dark urine, and a 25-pound weight loss at the time of admission. Liver function results were as follows: aspartate aminotransferase 43 U/L and alanine aminotransferase 53 U/L, alkaline phosphatase 442 U/L, albumin 3.2 g/dL, bilirubin total 2.5 mg/dL, and bilirubin direct 1.5 mg/dL. An endoscopic retrograde cholangiopancreatography showed strictures of the common bile duct suggestive of primary sclerosing cholangitis. A contrast-enhanced computed tomography scan (Figure 1) revealed an ill-defined, low-attenuating soft tissue mass in the porta hepatis with biliary duct dilatation, but no intrahepatic mass was identified. Based on these findings, a preoperative diagnosis of cholangiocarcinoma secondary to primary sclerosing cholangitis was made. The patient underwent a common bile duct resection, cholecystectomy, and Roux-en-Y hepaticojejunostomy. The mass was completely removed and sent to pathology for confirmatory diagnosis. Histologic sections of the left and the right bile ducts showed a histiocytic cell proliferation that consisted of mononuclear bean-shaped cells with cleaved nuclei and abundant cytoplasm admixed with eosinophils (Figure 2a, 2b). Sections of the adjacent liver showed variable bile duct proliferation, focal bridging and periductal fibrosis, and cholestasis. Immunohistochemistry revealed neoplastic cells that were positive for S100 (Figure 2c), CD1a (Figure 2d), CD68, CD14, and lysozyme. Electron microscopy of the tumor cells demonstrated the presence of Birbeck granules (Figure 2e) in the majority of histiocytes examined. These findings confirmed the diagnosis of LCH. The patient underwent a whole-body imaging to look for other areas of disease involvement, but the studies were negative. The patient received adjuvant chemotherapy with 5 courses of cladribine and was disease-free for 14 months after which she developed bacteremia due to a polymicrobial biliary infection from a chronic indwelling biliary drain. On admission, imaging studies did not reveal any evidence of malignancy. The patient went into septic shock, and despite therapeutic measures with antibiotics, vasopressor support, and volume resuscitation, she expired. An institutional review board waiver of consent was obtained for publishing the case report.

## DISCUSSION

LCH consists of a constellation of clinical presentations, which include Letterer-Siwe syndrome, a multisystem disease of young children characterized by cutaneous lesions containing infiltrates of Langerhans cells and a poor prognosis; eosinophilic granuloma, a solitary bone lesion; and Hand-Schuller-Christian disease that manifests as a characteristic triad of cranial bone lesions, diabetes insipidus, and exophthalmos (2). LCH can affect any organ or system in the body, but those more commonly affected are bone (80%), skin (33%), pituitary gland (25%), lungs (15%), liver (15%), the hematopoietic system (15%), lymph nodes (5%-10%), and the central nervous system excluding the pituitary gland (2%-4%) (4). The natural history of the disease is also extremely variable, ranging from a self-remitting lesion to a disease involving several organs with life-threatening consequences.

LCH involvement of the bile duct can occur, but it usually presents in the disseminated form of the disease and the prognosis is very poor (5,6). LCH of the bile duct can present with progressive destruction leading to sclerosing cholangitis and eventually to secondary biliary cirrhosis Table 1 (7,8,9). Primary LCH of the extrahepatic bile duct is very rare. Prior to our report, only one case has been reported in a 2.5-year-old female patient (10). A review of both cases showed that primary LCH of the bile duct presents as a solitary mass with clinical signs and symptoms of obstructive jaundice. Common sequelae of the mass appear to be sclerosing cholangitis, and both were diagnosed on a cholangiogram. Some reports suggest that the presence of sclerosing cholangitis, especially in children, should raise a suspicion for LCH (11,12). However, these studies included cases of disseminated LCH involving the hepatobiliary system, and involvement of the extrahepatic bile duct in these cases was rare. The key to a definitive diagnosis of LCH of the bile duct depends on the morphologic identification of abnormal proliferation of mononuclear cells with bean-shaped cleaved nuclei, which are derived from myeloid progenitor cells of the bone marrow (1). Similar to Langerhans cells located in the skin, they express histiocytic markers such as S100, CD1a, and CD68 and contain Birbeck granules, which are rod-shaped intracytoplasmic organelles best demonstrated on electron microscopy.

Treatment of LCH depends on the extent of the disease and can range from observation, surgery, and radiotherapy to multimodal chemotherapy. However, the precise treatment of primary LCH of the bile duct remains unknown due to limited data. Successful treatment with orthotropic liver transplantation was demonstrated by Finn and Jaffe (10) in their 2.5-year-old patient; however, the long-term effect of the procedure was not determined. Although our patient was successfully treated with surgical resection of the common bile duct with biliary bypass and post-adjuvant chemotherapy with cladribine, she died from complications of sepsis. Therefore, more insight is needed to determine the best treatment of LCH confined to the extrahepatic bile duct.

In conclusion, LCH confined to the extrahepatic bile duct is a rare phenomenon that is challenging to diagnose both by the clinician and by the pathologist. A high clinical suspicion should have been raised in our adult female patient with sclerosing cholangitis with a solitary biliary mass. Early diagnosis and treatment is needed to improve the clinical outcome.

## Figures and Tables

**Table 1:**
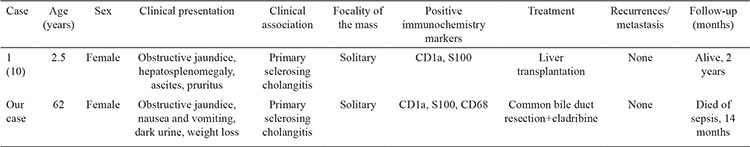
Reported cases of primary Langerhans cell histiocytosis of the biliary system
